# Epinephrine augments posttetanic potentiation in mouse skeletal muscle with and without myosin phosphorylation

**DOI:** 10.14814/phy2.13690

**Published:** 2018-05-02

**Authors:** Stephen Roy Morris, William Gittings, Rene Vandenboom

**Affiliations:** ^1^ Department of Kinesiology Brock University St. Catharines Ontario Canada

**Keywords:** Beta‐adrenergic, concentric twitch force, extensor digitorum longus, potentiation, regulatory light chains

## Abstract

Sympathetic tone may influence force potentiation, that is, the stimulation‐induced increase in skeletal muscle mechanical function associated with myosin phosphorylation, although the mechanism for this effect remains unknown. The purpose of this study was to examine the influence of epinephrine on concentric twitch force potentiation of wild‐type and skeletal myosin light‐chain kinase devoid mouse muscle (skMLCK ^−/−^). To this end, concentric twitch force was assessed before and after a potentiating stimulus (PS) to determine the peak and the duration of potentiation in the absence (−EPI) and presence (+EPI) of 1 *μ*mol/L epinephrine in both genotypes. Twitch force of wild‐type and skMLCK ^−/−^ muscles was increased by up to 31 and 35% and 18 and 23% in the −EPI and EPI conditions, respectively (all data *n* = 8, *P* < 0.05). In wild‐type muscles, the PS increased RLC phosphorylation from 0.14 ± 0.05 (rest) to 0.66 ± 0.08 mol phos mol RLC; by 480 sec RLC phosphorylation had returned to baseline (all data *n* = 4 each time point, *P* < 0.05). Neither resting nor peak levels of RLC phosphorylation were altered by +EPI, although the duration of RLC phosphorylation was prolonged. In skMLCK ^−/−^ muscles, RLC phosphorylation was not elevated above constituent levels by stimulation in either the −EPI or +EPI condition. Thus, given the similarity in potentiation responses between genotypes our data suggest that the influence of epinephrine on potentiation was independent of skMLCK catalyzed phosphorylation of the RLC, although the clinical significance of this pathway for skeletal muscle function remains to be identified.

## Introduction

Recognized in the literature for over 100 years (see Lee (1907)) the potentiation of isometric twitch force has long been considered a fundamental property of the mammalian fast, but not slow, muscle phenotype (Buller et al. 1981; Close and Hoh 1968, 1969). The transient increase in isometric or dynamic muscle force of rodent fast skeletal muscle types studied in vitro has been temporally correlated with the phosphorylation of the myosin regulatory light chain (RLC) (Caterini et al. 2011; Grange et al. 1995, 1998; Klug et al. 1982; Manning and Stull 1982; Moore and Stull 1984; Moore et al. 1990; Palmer and Moore 1989; Vandenboom et al.^1995^, ^1997^; Xeni et al. 2011). On the other hand, rodent fast muscles without the ability to phosphorylate the RLC display a much reduced, but persistent, potentiation (Tubman et al. 1996, 1997; Zhi et al. 2005). Together, these studies suggest that although other mechanisms may exist (e.g., Rassier et al. 1999; Smith et al. 2013), phosphorylation of the myosin RLC modulates contractile function to potentiate force of rodent fast twitch skeletal muscle types.

Phosphorylation of the myosin RLC is catalyzed by myosin light‐chain kinase (skMLCK) (Stull et al. 2011). This phosphotransferase activity is regulated via a Ca^2+^ and calmodulin signaling cascade in which the myoplasmic Ca^2+^ levels regulating contraction fully activate skMLCK (Ryder et al. 2007). Dephosphorylation of the myosin RLC is accomplished via phosphatase enzyme activity which is unregulated and maintained at constitutive levels only. The rapid activation and slow deactivation kinetics of skMLCK, when combined with low phosphatase activity, provides a “molecular memory” that modulates contractile protein performance during intermittent activity (Ryder et al. 2007). The structure–function explanation for how RLC phosphorylation modulates contractile performance has recently been reviewed (Vandenboom 2017).

An important aspect of organismal function is the influence that extracellular signaling may have skeletal muscle contractile performance. An example of this is the adrenergic mediated “fight‐or‐flight” response, a signaling axis that may involve RLC phosphorylation‐mediated potentiation. For example, Decostre et al. (2000) used mouse EDL muscle to show that *β*‐agonist stimulation prolonged stimulation‐induced increases in both RLC phosphorylation and isometric twitch potentiation. These results were interpreted as an epinephrine‐mediated decrease in MLCP activity that prolonged both RLC phosphorylation and potentiation. Although entirely plausible, the inherent inability to stimulate wild‐type muscles without concomitant RLC phosphorylation means that skMLCK contributions to this effect cannot be excluded entirely.

The purpose of this study was to clarify the mechanism by which epinephrine enhances potentiation. To do this we assessed the effect of 1 *μ*mol/L epinephrine on EDL muscles from both wild‐type and skMLCK devoid (skMLCK^−/−^) mice, an approach which allowed us to assess how the absence of skMLCK‐mediated RLC phosphorylation altered epinephrine effects. We hypothesized that, in the absence of skMLCK, the influence of epinephrine on these respective responses would be blunted, thus supporting the idea that *β*‐adrenergic stimulation influences contractile activity via skMLCK activity. Because potentiation may be the normal or optimal state for working skeletal muscle in vivo (Tsianos et al. 2012; Tsianos and Loeb 2014), identification of the precise pathway for this outcome may be important for developing interventions aimed at improving muscle function compromised either by age or by disease.

## Methods

All procedures utilized in this study received ethical approval from the Brock University Animal Care Committee. Adult male C57BL/6 wild‐type mice (age 3–6 months) were ordered from Charles River Labs (St Constant, QC) and skMLCK^−/−^ mice (age 2–5 months) were obtained from our own breeding colony at Brock University. Details regarding the generation and characterization of skMLCK^−/−^ have been previously presented (Gittings et al. 2011, 2015; Zhi et al. 2005). Descriptive data for the mice used in this study are compiled in Table 1. On the day of an experiment, mice were anesthetized with an intraperitoneal injection of sodium pentobarbital (60 mg/kg body mass) diluted with 0.9% saline in a 1 ml syringe. The *extensor digitorum longus* (EDL) muscle was then surgically excised and mounted in a jacketed vertical organ bath containing continuously oxygenated Tyrode's physiological solution (Lannergren et al. 2000) and maintained at 25°C. The mouse EDL muscle is composed almost exclusively of type IIb and IIx fiber types (Smith et al. 2013) and as such is ideal for studying potentiation phenomena. Moreover, its small size enables it to remain metabolically viable for long periods of time at 25°C (Barclay 2005). Muscle stimulation was applied using flanking platinum electrodes, provided by a model 701B biphasic stimulator (Aurora Scientific Inc., Aurora, ON) with voltage set to 1.25 times the threshold required to active all fibers and elicit maximal isometric twitch force. A 30 min equilibration period followed during which the optimal length for maximal isometric twitch force was determined (L_o_). During experiments, muscle length and force data were monitored via LINUX software and controlled by a dual‐mode servomotor (Model 305B, Aurora Scientific Inc., Aurora, ON). All experiments were collected at 2,000 Hz and saved to computer for further analysis (ASI 600a software).

**Table 1:** Masses of wild‐type and skMLCK^−/−^ mice used in experiments

Genotype	Contractile	Biochemical
Wild‐type	25.8 ± 0.6	28.3 ± 0.9
skMLCK^−/−^	19.4 ± 0.5^a^	22.4 ± 3.0^a^

Values are means ± SEM expressed in grams. *N* = 5 for muscles used in contractile experiments and *n* = 6–12 for muscles used in biochemical experiments.

a skMLCK^−/−^ mass less than wild‐type mass (*P* < 0.05).

### Experimental protocol

**Figure 1:**
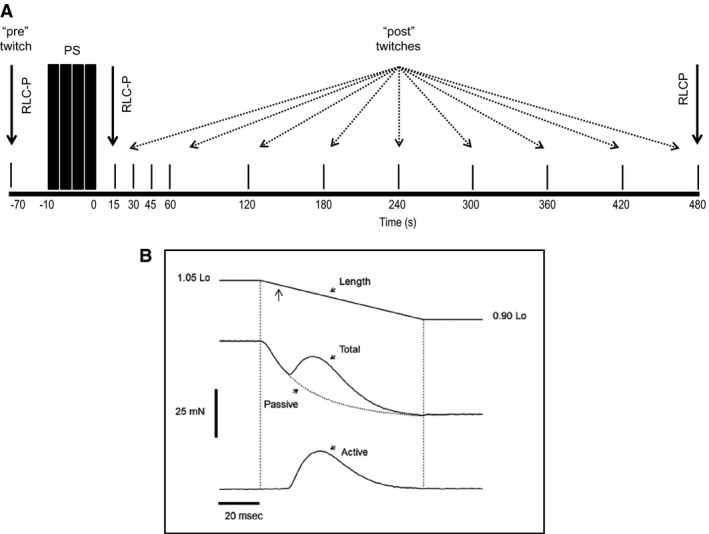
(A) Experimental timeline for muscles of both genotypes (wild‐type and skMLCK ^−/−^) and conditions (control and 1 *μ*mol/L epinephrine). Each experiment began with a series of three isovelocity twitches which were averaged to produce an unpotentiated (control) twitch. Muscles shortened from 1.05 to 0.90 of optimal length (Lo) at 0.30 *V*_max_ (Gittings et al. 2011) during each of these events. The stimulus pulse was timed so that peak concentric force coincided with 1.00 Lo. Sixty seconds after the last twitch in that series a potentiating stimulus (PS) was applied with muscle length fixed at 1.00 Lo. The PS consisted of four (4) brief (400 msec) but high‐frequency (100 Hz) volleys within a 10 sec time window. A series of isovelocity twitches were then elicited at various times after the PS to capture the peak and the duration of concentric force potentiation. For each muscle of each genotype the control (no epinephrine) paradigm was conducted before the experimental (with epinephrine) paradigm. A 30 min equilibration period separated the two series of experiments. The three times at which muscles of each genotype and in each condition were frozen for RLC phosphorylation level are indicated by vertical solid arrows. Note that each vertical line represents an “active” twitch event (i.e., passive not shown) (not to scale). (B) Method for obtaining active concentric twitch force during isovelocity ramp shortening. Representative records for length, total force, passive force, and calculated active force (total – passive) are shown. Example is from a WT muscle in control condition, but the procedure was the same for all contractions in all experiments. Calibration bars for force and time are shown; starting and ending muscle lengths also indicated with vertical dashed lines indicating start and end of length ramp. Vertical arrow denotes the stimulus pulse triggering the twitch event. See Methods for more details. The parameters of the shortening ramp were identical for all muscles and conditions in terms of amplitude and speed (i.e., 0.15 L_o_ and 30% *V*_max_, respectively). In the example shown the shortening ramp was completed in ~80 msec.

The experimental protocol for determining the influence of epinephrine incubation on concentric twitch force production is shown in Figure 1. Mean unpotentiated (control) concentric twitch force was calculated from three consecutive twitches at 20 sec intervals; the central twitch of this series was elicited 60 sec before the start of the PS. The PS consisted of four brief (400 msec) but high‐frequency (100 Hz) volleys within a 10 sec time window, a protocol that produces near maximum twitch potentiation with little fatigue in mouse EDL muscle. Following this, concentric twitch force was measured at selected times for up to 8 min in order to capture the peak and the duration of potentiation. The ratio of post‐ to pre‐PS force was used to assess potentiation magnitude for each time point. Because we used both wild‐type and skMLCK^−/−^ muscles we were able to quantify the interaction of epinephrine and RLC phosphorylation on twitch force potentiation between or across genotypes. Additionally, muscles were frozen before as well as 15 and 480 sec after the PS for subsequent determination of RLC phosphorylation.

### Shortening ramps

Concentric twitch force was produced by stimulating muscles to contract while shortening at a steady rate. To achieve this, a “ramp” change in length was applied to the muscle which allowed it to shorten from 1.05 to 0.90 L_o_. During these isovelocity ramps a single stimulus pulse was delivered shortly after the start of shortening so that peak concentric force occurred around 1.00 Lo. The rate of ramp was equivalent to 30% *V*_max_ for all twitches and muscles, a value scaled to a shortening speed of 9.8 fL sec^−1^ for mouse EDL of both genotypes at 25°C (Brooks and Faulkner 1988). To calculate active concentric force required paired “passive” and “active” events; during the passive event the muscle received a shortening ramp but was not stimulated while during the active event the muscle was stimulated during the shortening ramp. As shown in Figure 1B, active concentric force representative of a time or condition was obtained by subtracting the “passive” force response from the matching “total” force response. The rate of concentric force development (+dF/dt) and concentric force relaxation (−dF/dt) were determined for each twitch by taking the first derivative of the active force records. The time between the start of twitch force development and the attainment of peak force (i.e., the time to peak tension, TPT) and the elapsed time from the peak of twitch tension to 50% of the peak (i.e., the half relaxation time, ½ RT) of each twitch in each condition and genotype were also recorded.

### Epinephrine preparation

Experimental concentrations of epinephrine were determined based on work by Decostre et al. (2000) and Andersson et al. (2012). On the day of an experiment a fresh stock solution of 1 mmol/L epinephrine was prepared using epinephrine hydrochloride (E4642 SIGMA). After the control (−EPI) experiments were concluded a volume of this stock solution was added to the muscle bath to yield a 1 *μ*mol/L concentration of epinephrine. Each muscle was allowed a 30 min incubation period before the + EPI series of experiments was performed. Control experiments were performed to determine the time‐dependent influence of epinephrine. In these experiments a subset (*n* = 3) of muscles was incubated for 30 min in 1 *μ*mol/L epinephrine after which the increased twitch force was tracked for 10 min. It was observed that twitch force varied by no more than 3% (data not shown) from the first to the last twitch in this time window, suggesting that epinephrine effects on PTP were relatively constant.

### Myosin phosphorylation

On the day of analysis muscle samples were removed from −80°C storage and, following denaturization by dithiothreitol (DTT) and trichloroacetic acid (TCA), were homogenized by mortar and pestle and prepped for gel electrophoresis. After centrifugation at 2000 rpm for 2 min, the supernatant was poured off leaving a ‘pellet’. The remaining tissue pellet was then washed using ethyl ether to remove excess TCA. Following this, the pellet was resuspended in a urea‐based sample buffer (at 30 volumes per 1 mg tissue: 8 mol/L Urea, 1.83 mL; Urea Gel Buffer, 167 *μ*L; 0.5 mol/L DTT, 40 *μ*L; Saturated Sucrose, 100 *μ*L; 0.2% Bromphenol Blue, 40 *μ*L; 0.4 mol/L EDTA, 1 *μ*L) and urea crystals were added, as necessary, to achieve complete saturation and solubilize the RLC of each sample. Samples were pipetted into separate ‘wells’ of a BIO RAD minigel electrophoresis apparatus (10 *μ*L per sample) containing a 30% polyacrylamide gel with the addition of glycerol for density. Gels were run for 85 min at 400 V, and then electrophoretically transferred (60 min at 25 V) to a nitrocellulose membrane using a nonconducting gel‐membrane cassette submerged in transfer buffer. After transfer, the membranes were washed with Tris buffered saline + Tween 20 (TBST), followed by incubation in blocking buffer for 1 h. Lastly, the nitrocellulose gels were incubated with primary antibody (1:7500; skMLCK polyclonal antibodies provided by Dr. James Stull, UT Southwestern, Dallas TX) and then stored overnight at ~4°C. The next day the membranes were washed with TBST to remove the primary antibody, immediately followed by incubation with secondary goat anti‐rabbit IgG‐horseradish peroxidase (1:10000; Santa Cruz Biotechnology, Inc.) in TBST for 1 h at room temperature. The membranes were then incubated with a detection buffer (enhanced chemiluminescence prime) for 3 min before being rinsed with dH_2_0, drained, and placed in a FLUORCHEM 5500 for exposure of the membrane and photoanalysis.

### Statistical analysis

Data were initially screened for outliers, and checked for statistical assumptions of normal distribution and equality of variances after which each dataset was analyzed separately using Prism 6 Graphpad. Within genotype factors were analyzed using a two‐way repeated measures ANOVA for each condition followed by a Holm‐Sidak's correction. Within genotype influences of epinephrine were analyzed using a two‐way repeated measures ANOVA followed by a Sidak's multiple comparisons test. Between genotype factors were analyzed using a two‐way repeated measures ANOVA for each condition followed by a Sidak's correction. Differences in peak tetanic forces during PS between genotypes were analyzed using *t*‐tests followed by a Sidak's correction (*P *<* *0.05). To determine the effect of stimulation on RLC phosphorylation a two‐way ANOVA was conducted followed by a Dunnet's test for significance over time. In addition, multiple *t*‐tests were used to determine the effect of epinephrine on RLC phosphorylation in WT muscles at each time point. Unless noted otherwise, all data are reported as the mean ± SEM (*n* = 8) with *P* < 0.05 used as significant difference.

## Results

**Figure 2:**
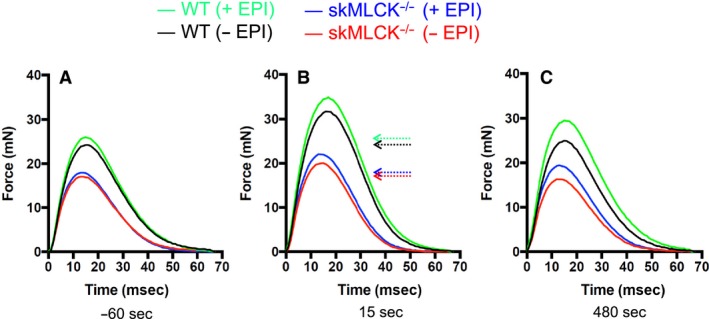
Active concentric twitch force records of extensor digitorum longus muscles (in vitro, 25°C) from all wild‐type and skMLCK ^−/−^ mice used in these experiments. (A) Unpotentiated (control) twitches obtained before the PS. (B) Potentiated twitches obtained 15 sec after the PS. (C) Twitches obtained 480 sec after the PS. These records show the influence of genotype and epinephrine (compare within panels) as well as the effect of time (compare between panels) on concentric twitch force potentiation. Note that muscles from skMLCK ^−/−^ mice generated less force at each time point and condition and potentiated less than their wild‐type counterparts. The influence of epinephrine on concentric twitch force amplitude was similar at each time point in each genotype, however. Records also show similarity of change to twitch time course were similar across conditions and genotype. Each trace is the mean response of eight muscles at each time point. Horizontal arrows in the middle panel reference unpotentiated levels for each genotype and condition. Note that although subtraction of the passive tension present during the unstimulated from the stimulated record “normalized” the active force record, an influence on potentiation may still have been present and must be considered a RLC phosphorylation independent mechanism.

Concentric twitch force and RLC phosphorylation were assessed in wild‐type and skMLCK^−/−^ muscles to test the mechanism by which epinephrine influences potentiation. Absolute data describing mean concentric twitch force for untreated (−EPI) and treated (+EPI) before and after the PS are presented in Table 2 for both genotypes. Records of twitch force collected at time points corresponding to RLC phosphorylation measurements in both genotypes are shown in Figure 2.

**Table 2:** Effect of epinephrine on peak concentric twitch force before and at selected times after a PS

Con	−60	15	30	45	60	120	240	360	480
Wild‐type									
−EPI	24.1 ± 2.4	31.6 ± 3.4^a^	29.6 ± 3.3^a^	29.8 ± 3.3^a^	29.4 ± 3.2^a^	28.4 ± 3.0^a^	26.6 ± 2.8^a^	25.5 ± 2.6^a^	24.9 ± 2.5
+EPI	25.6 ± 2.4	34.6 ± 3.3^a^	32.5 ± 3.2^a^	32.7 ± 3.3^a^	32.6 ± 3.2^a^	31.7 ± 3.1^a^	30.3 ± 3.0^a^	29.8 ± 2.9^a^	29.1 ± 2.7^a^
Relative	1.06 ± 0.10^b^	1.09 ± 0.11^b^	1.10 ± 0.11^b^	1.10 ± 0.11^b^	1.11 ± 0.11^b^	1.11 ± 0.11^b^	1.14 ± 0.10^b^	1.17 ± 0.11^b^	1.17 ± 0.11^b^
skMLCK^−/−^									
−EPI	17.6 ± 1.5	20.7 ± 1.7^a^	19.3 ± 1.6^a^	19.2 ± 1.6^a^	18.8 ± 1.5^a^	18.0 ± 1.4	17.0 ± 1.2	16.5 ± 1.2	16.7 ± 1.3
+EPI	18.4 ± 1.5	22.4 ± 1.8^a^	20.9 ± 1.8^a^	20.9 ± 1.7^a^	20.6 ± 1.6^a^	20.3 ± 1.6^a^	19.8 ± 1.5^a^	19.6 ± 1.5^a^	19.7 ± 1.5^a^
Relative	1.04 ± 0.09	1.08 ± 0.09^b^	1.08 ± 0.09^b^	1.09 ± 0.09^b^	1.11 ± 0.09^b^	1.13 ± 0.09^b^	1.16 ± 0.09^b^	1.19 ± 0.09^b^	1.18 ± 0.09^b^

Values are means ± SEM (*n *=* *8) expressed in mN. The relative value at the bottom of each column was calculated by dividing the –EPI by the + EPI value at each time point for each genotype. Absolute values for WT muscles were greater than for skMLCK^−/−^ in each condition and at each time point. Untreated muscles (‐ EPI); epinephrine‐treated muscles (+ EPI).

a Indicates postvalue significantly greater than prevalue within each genotype *(P < 0.05)*.

b Indicates + EPI condition greater than –EPI condition at that time point within each genotype. Note that skMLCK^−/−^ muscles did not display any potentiation of peak concentric force beyond 60 s after the PS unless epinephrine was present.

### Effect of stimulation and epinephrine on wild‐type and skMLCK^−/−^muscles

**Figure 3:**
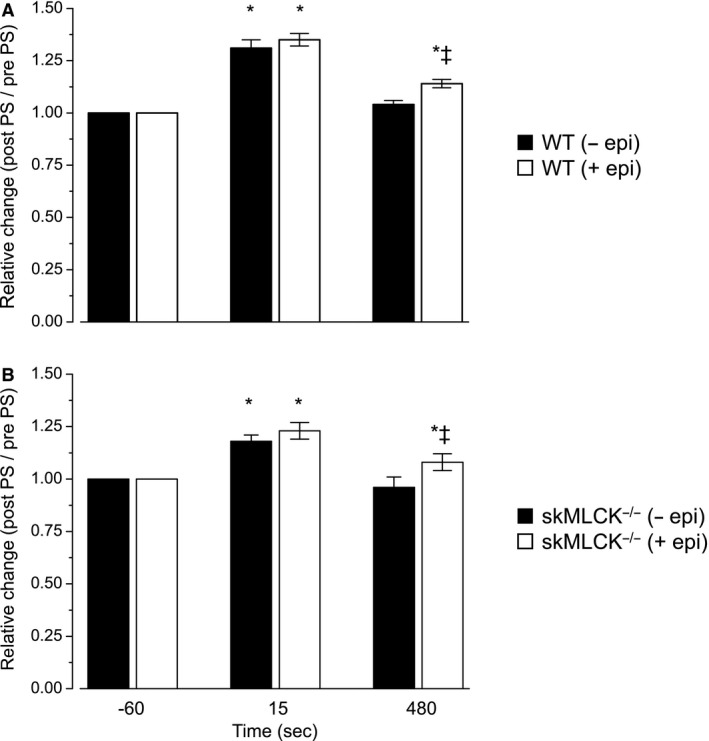
Summary data showing relative concentric twitch force (post‐PS/pre‐PS) in WT (A) and skMLCK ^−/−^ (B) (both *n* = 8). Closed bars, Control; Open bars, Epinephrine Treated. * indicates that post‐PS value is significantly greater than unpotentiated control value. ☨Value for epinephrine (+EPI)‐treated muscles was higher than for untreated muscle (−EPI) within each genotype. Only those time points at which RLC phosphorylation was determined are compared in this plot.

**Figure 4:**
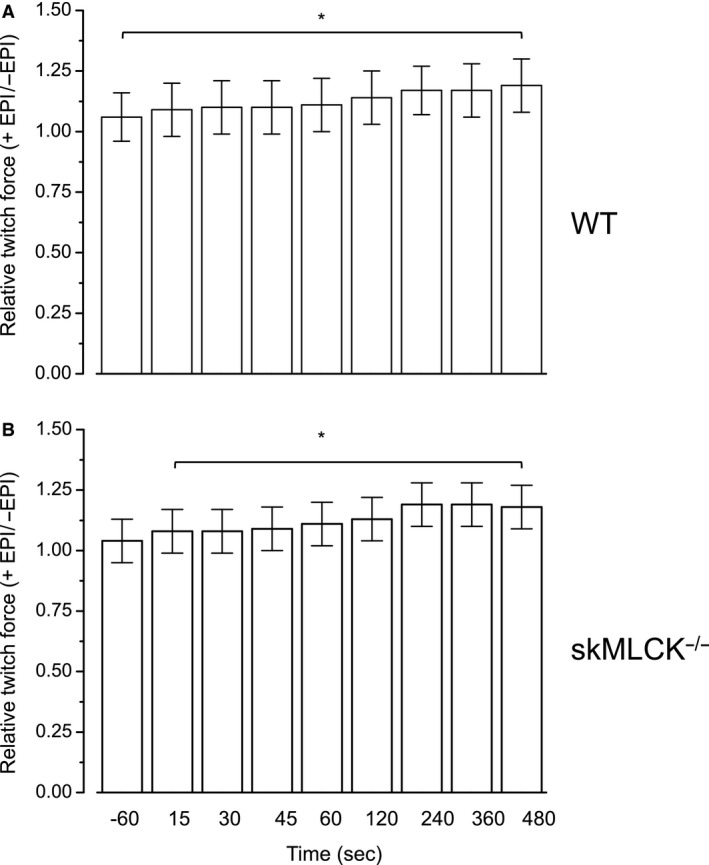
Summary data showing influence of epinephrine for select time points before and after the PS within wild‐type (A) and skMLCK ^−/−^ (B) muscles. Value at each time point was obtained by dividing control (−EPI) value by epinephrine treatment (+EPI) value at each time point (*n* = 8 each time point). * denotes + EPI value different from –EPI value for that genotype (*P* < 0.05). In both wild‐type and skMLCK ^−/−^ muscles, + EPI increased peak concentric twitch force potentiation by ~13% when data from all post‐PS time points are collapsed. Plot based on data in Table 2.

Peak twitch force of untreated WT muscles was increased for up to 6 min following the PS. For example, concentric force was increased by 31 ± 4 and by 6 ± 2% when determined at 15 and 360 sec, respectively. Epinephrine increased the magnitude of twitch force potentiation caused by the PS at all time points; the interaction of potentiation and epinephrine on mean concentric twitch force of wild‐type muscles is summarized in Figures 3A and 4A. Peak twitch force of untreated skMLCK^−/−^ muscles was increased by the PS although this change was not as great and did not last as long as that observed in wild‐type muscles. For example, concentric force was increased by 18 ± 3 and 7 ± 3% when determined at 15 and 60 sec, respectively; by 120 sec, however, twitch forces were not different from unpotentiated levels. In contrast to wild‐type muscles, epinephrine did not influence unpotentiated twitch forces of skMLCK^−/−^ muscles. Similar to wild‐type muscles, however, epinephrine increased both the magnitude and duration of the twitch force potentiation caused by the PS. The combined effect of epinephrine and potentiation (i.e., the interaction) on mean concentric twitch force of skMLCK^−/−^ muscles is summarized in Figures 3B and 4B.

### Effect of stimulation and epinephrine on twitch kinetics of wild‐type and skMLCK^−/−^muscles

Absolute data describing the rate of concentric force development (+dF/dt) and relaxation (−dF/dt) of wild‐type muscles are shown in Table 3A. These data show that the +dF/dt was increased at all time points following the PS but was highest in the first 120 sec after the PS (range: 28–39%); by 480 sec after the PS the +dF/dt was still increased by 9% relative to before the PS. Epinephrine increased the unpotentiated as well as the potentiated +dF/dt at most time points (i.e., by 14 and 17–37%, respectively). In contrast, epinephrine did not alter unpotentiated values for −dF/dt, although it did increase the −dF/dt of potentiated twitches in the first 60 sec after the PS (range: 12–25%). Absolute data describing +dF/dt and −dF/dt of skMLCK^−/−^ muscles are shown in Table 3B. The +dF/dt of twitches was increased for up to 30 sec after the PS (range: 8–20%). Epinephrine increased unpotentiated +dF/dt by ~12% but had only a limited effect on potentiated +dF/d*t* (i.e., increased 7–16% increase over first 360 sec). The −dF/dt of skMLCK^−/−^ muscles was increased for the first 60 sec after the PS (range: 8–17%) before returning to pre‐PS levels by ~ 180 sec. Epinephrine did not alter unpotentiated −dF/dt or potentiated −dF/dt during the first 120 sec after the PS. After this time point, however, epinephrine did increase potentiated –dF/dt compared to control values.

**Table 3:** (A) Effect of epinephrine on twitch force kinetics of wild‐type muscles before and at selected times after a PS. (B) Effect of epinephrine on concentric force kinetics of skMLCK^−^/^−^ muscle before and at selected times after a PS

(A)									
Cond	−60	15	30	45	60	120	240	360	480
+dF/dt									
−EPI	2.25 ± 0.29	3.07 ± 0.32^a^	3.01 ± 0.35^a^	3.00 ± 0.36^a^	2.92 ± 0.37^a^	2.85 ± 0.34^a^	2.64 ± 0.34^a^	2.50 ± 0.33^a^	2.43 ± 0.32^a^
+EPI	2.56 ± 0.32	3.44 ± 0.34^a^	3.33 ± 0.34^a^	3.33 ± 0.35^a^	3.29 ± 0.34^a^	3.20 ± 0.34^a^	3.07 ± 0.34^a^	3.03 ± 0.34^a^	2.96 ± 0.32^a^
Relative	1.14 ± 0.13^b^	1.12 ± 0.13^b^	1.12 ± 0.10^b^	1.11 ± 0.11^b^	1.13 ± 0.11^b^	1.13 ± 0.11^b^	1.16 ± 0.11^b^	1.21 ± 0.12^b^	1.22 ± 0.12^b^
−dF/dt									
−EPI	−1.39 ± 0.17	−1.71 ± 0.19^a^	−1.62 ± 0.18^a^	−1.61 ± 0.18^a^	−1.53 ± 0.17^a^	−1.46 ± 0.16	−1.38 ± 0.17	−1.35 ± 0.16	−1.30 ± 0.15
+EPI	−1.36 ± 0.13	−1.75 ± 0.17^a^	−1.66 ± 0.16^a^	−1.65 ± 0.16^a^	−1.55 ± 0.18^a^	−1.48 ± 0.16^a^	−1.45 ± 0.16^a^	−1.40 ± 0.15	−1.41 ± 0.14
Relative	0.98 ± 0.08	1.02 ± 0.10	1.02 ± 0.10	1.02 ± 0.09	1.01 ± 0.10	1.01 ± 0.09	1.05 ± 0.10^b^	1.04 ± 0.10	1.08 ± 0.10^b^

(B)									
Cond	−60	15	30	45	60	120	240	360	480
+dF/dt									
−EPI	2.16 ± 0.21	2.52 ± 0.22^a^	2.51 ± 0.25^a^	2.41 ± 0.23^a^	2.32 ± 0.23	2.24 ± 0.21	2.09 ± 0.21	2.04 ± 0.22	2.02 ± 0.23
+EPI	2.42 ± 0.24	2.78 ± 0.28^a^	2.71 ± 0.28^a^	2.65 ± 0.29^a^	2.58 ± 0.27	2.55 ± 0.27	2.47 ± 0.25	2.45 ± 0.25	2.46 ± 0.28
Relative	1.12 ± 0.10^b^	1.10 ± 0.10^b^	1.08 ± 0.11^b^	1.10 ± 0.10^b^	1.11 ± 0.10^b^	1.14 ± 0.11^b^	1.18 ± 0.11^b^	1.23 ± 0.11^b^	1.19 ± 0.13^b^
−dF/dt									
−EPI	−1.02 ± 0.09	−1.19 ± 0.12^a^	−1.13 ± 0.10^a^	−1.11 ± 0.10^a^	−1.09 ± 0.09	−1.05 ± 0.09	−0.98 ± 0.08	−0.96 ± 0.07	−0.98 ± 0.10
+ EPI	−1.08 ± 0.08	−1.26 ± 0.11^a^	−1.18 ± 0.10^a^	−1.20 ± 0.10^a^	−1.16 ± 0.10^a^	−1.11 ± 0.09	−1.11 ± 0.09	−1.08 ± 0.07	−1.09 ± 0.08
Relative	1.06 ± 0.07	1.06 ± 0.07	1.03 ± 0.08	1.08 ± 0.08^a^	1.06 ± 0.09	1.06 ± 0.08	1.13 ± 0.08^b^	1.11 ± 0.06^b^	1.11 ± 0.08^b^


Values are means ± SEM (*n *=* *7) expressed in mN ms^1^. The relative value at the bottom of each column was calculated by dividing the −EPI by the + EPI value at each time point for each genotype.

Con, condition; −EPI, untreated muscles; +EPI, epinephrine‐treated muscles.

a Indicates post‐PS value significantly greater than pre‐PS value within each genotype (*P* < 0.05).

b Indicates epinephrine significantly enhanced value at that time point within each genotype.

### Tetanic forces during PS

Values for peak isometric force obtained of wild‐type and skMLCK^−/−^ muscles are shown in Table 4. Although peak isometric forces of skMLCK^−/−^ muscles was lower than that for wild‐type muscles, the influence of epinephrine on this measure was similar in both genotypes.

**Table 4:** Peak tetanic forces of wild‐type and skMLCK^−/−^ muscles with and without epinephrine

Genotype	‐EPI	+EPI	Relative
Wild‐type	226.3 ± 19.3	248.6 ± 21.5	1.10
skMLCK^−/−^	177.0 ± 14.0^a^	199.1 ± 15.1^a^	1.12

Values are means ± SEM (*n *=* *8) expressed in mN. Value represents the tetanic force determined from the first volley of each potentiating stimulus (PS). Relative (+ EPI/− EPI) difference calculated in column at right represents influence of epinephrine within each genotype.

−EPI, untreated muscles; + EPI, epinephrine‐treated muscles.

a Indicates that skMLCK^−/−^ value is significantly different than wild‐type value.

### Effect of stimulation and epinephrine on twitch time course of wild‐type and skMLCK^−/−^muscles

In general, the time course of unpotentiated twitches was not altered by epinephrine. For example, the TPT and ½RT of unpotentiated WT muscles were 13.8 ± 0.8 and 26.1 ± 2.1 msec, respectively. Moreover, although the TPT was increased immediately after the PS this effect quickly dissipated. On the other hand, the ½RT of potentiated twitches was not altered compared to unpotentiated twitches at any time point after the PS. No influence of epinephrine was found for the TPT or ½ RT of unpotentiated or potentiated twitches. No genotype‐dependent differences in unpotentiated twitch time course were detected. For example, the TPT and ½ RT of unpotentiated twitches were 13.7 ± 0.6 and 26.4 ± 2.2 msec, respectively. Similar to wild‐type muscles, the TPT of potentiated twitches was increased ~10% immediately after the PS, an effect that quickly dissipated while the ½RT of potentiated twitches was not altered at any time point. Similar to wild‐type muscles, the presence of epinephrine had little effect on either the TPT or the ½RT of unpotentiated or potentiated twitches in this genotype.

### Effect of stimulation and epinephrine on myosin RLC Phosphorylation in wild‐type and skMLCK^−/−^muscles

**Figure 5:**
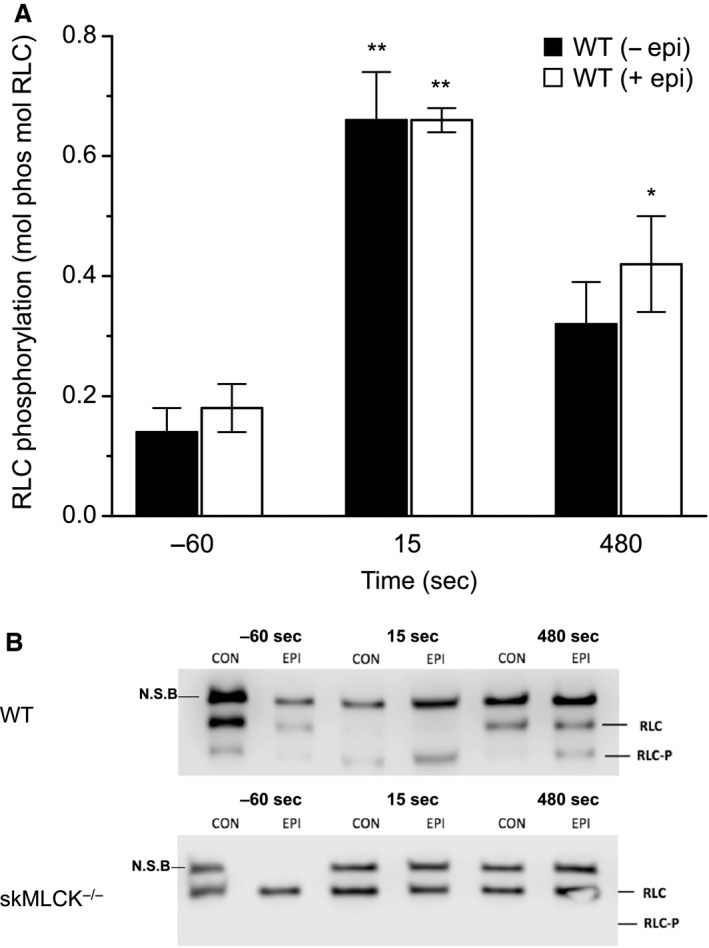
RLC phosphorylation in EDL muscles from wild‐type and skMLCK ^−/−^ mice. (A) Effect of time and epinephrine treatment on RLC phosphorylation in EDL muscles from wild‐type mice. Values are shown for rest (60 sec before PS) as well as ~15 and 480 sec after the PS (all date *n *=* *4). No differences in RLC phosphorylation were detected between conditions (−EPI vs. + EPI) at any time point. Only wild‐type data are shown. **Indicates post‐PS value is significantly higher than pre‐PS value in that condition. * Indicates 480 sec value is significantly lower than 5 sec value but remains significantly elevated above resting value in that condition. Note that each time point example is from a different blot (three different blots total). (B) Mosaic of urea/glycerol PAGE blots showing differences in RLC phosphorylation in *WT* (top panel) and skMLCK ^−/−^ (bottom panel) muscles from both control (CON) and epinephrine (EPI) conditions. Each lane represents a different sample and was typically characterized by three separate bands: a nonspecific binding band (NSB), a nonphosphorylated and a monophosphorylated band (RLC and RLC‐P, respectively). The RLC‐P band was identified based on the specific increase in protein migration relative to the RLC band. Note that although some blots did display detectable levels for RLC phosphorylation (~5%) in skMLCK ^−/−^ muscles, this was not evident in the blot shown. *WT* western blots (−60 sec, 15 sec, and 480 sec) display three separate blots for each condition, where the image for −60 sec condition is an example for the absence of the NSB. EDL, extensor digitorum longus.

Summary data for RLC phosphorylation of both genotypes are shown in Figure 5A with example western blots shown in Figure 5B. In wild‐type muscles, resting RLC phosphorylation was similar in the absence and presence of epinephrine (i.e., 0.14 ± 0.05 and 0.18 ± 0.03 mol phos mol RLC, respectively). In addition, the increase in RLC phosphorylation caused by the PS was similar in untreated and treated muscles (i.e., 0.66 ± 0.08 and 0.66 ± 0.02 mol phos mol RLC, respectively). RLC phosphorylation determined in muscles frozen 480 sec after the PS was similar and diminished from peak values in both untreated and treated muscles (i.e., 0.32 ± 0.06 and 0.42 ± 0.08 mol phos mol RLC, respectively). Only in epinephrine‐treated muscles was this final value elevated above rest, however. By contrast, no RLC phosphorylation was detected at any stage of any experiment in skMLCK^−/−^ muscles.

## Discussion

The purpose of this study was to test the idea that epinephrine influences posttetanic potentiation of concentric twitch force in mouse EDL muscle by increasing RLC phosphorylation. Our main finding was that, although wild‐type muscles consistently displayed more potentiation than did skMLCK^−/−^ muscles, epinephrine treatment had comparable effects on potentiation in both genotypes. Moreover, epinephrine had no effect on RLC phosphorylation levels in either genotype. Thus, we reject the hypothesis that epinephrine enhances potentiation via an effect on skMLCK catalyzed phosphorylation of the RLC in the wild‐type fast muscles phenotype.

Although wild‐type and skMLCK^−/−^ mice have been compared previously, this is the first study to compare the time course of concentric twitch force potentiation in both genotypes. A novel aspect of this study is thus our data showing that skMLCK ablation reduces both the magnitude and duration of concentric twitch force potentiation. Indeed, at a time when concentric twitch force of skMLCK^−/−^ muscles had returned to baseline in the control condition (i.e., 120 sec) wild‐type muscles still displayed considerable potentiation. Thus, our data suggest that a more robust potentiation of concentric force is provided when the RLCs are phosphorylated compared to when they are not (see also Bowslaugh et al. 2016; Bunda et al. 2018; Gittings et al. 2017; Gittings et al. 2018; Vandenboom et al. 2013). Because peak values for RLC phosphorylation were similar in both the untreated and treated conditions, it seems unlikely that epinephrine influences skMLCK activity in wild‐type muscles. On the other hand, epinephrine did prolong (i.e., 480 sec) levels of RLC phosphorylation in wild‐type muscles compared to rest, suggesting an influence on MLCP activity as originally suggested by Decostre et al. (2000). This influence did not, however, differentially influence potentiation of concentric twitch force as the relative increase between the epinephrine‐treated and untreated muscles was similar in both genotypes. Thus, the influence of epinephrine to enhance potentiation was uncoupled from skMLCK catalyzed RLC phosphorylation. A regression analysis comparing the relative difference in twitch force between wild‐type and skMLCK^−/−^ muscles with and without epinephrine showed a very strong correlation between control and epinephrine‐treated muscles (*r* ^*2*^ = 0.90) of both genotypes (data not shown). Although the mechanism for this effect remains unknown, this analysis supports the notion that the influence of epinephrine on concentric twitch force was mediated by a RLC phosphorylation independent pathway.

Work by Andersson et al. (2012) showed that administration of the *β*‐agonist isoproterenol increased force of wild‐type muscles but had no effect on muscles from ryanodine receptor type 1 (RyR1) knockout mice. These results indicate that the influence of *β*‐agonist stimulation on isometric twitch force is mediated via the RyR1 channels and increased Ca^2+^ release. Assuming that this cascade was not altered by skMLCK ablation, this mechanism could provide a RLC phosphorylation independent pathway for potentiation and account for why epinephrine increased potentiation equally in both wild‐type and skMLCK^−/−^ muscles. Consistent with this hypothesis there is little evidence to conclusively support the idea that MLCP shares a similar control mechanism with the glycogen‐bound protein phosphatase (PP1‐G) (Gailly et al. 1996; Johnson et al. 1996; Egloff et al. 1997). This makes it unlikely that this mechanism was responsible for the increase in RLC phosphorylation observed by Decostre et al. (2000).

A critical aspect of this study was our use of the skMLCK^−/−^ genotype as a negative control for the absence of RLC phosphorylation following stimulation. Indeed, in contrast to the wild‐type genotype, we detected only constituent, low levels of RLC phosphorylation in skMLCK^−/−^ muscles with no stimulation‐induced increases. Indeed, poststimulus values for RLC phosphorylation reported from studies using skMLCK^−/−^ muscles are similar to resting values in wild‐type muscles (Zhi et al. 2005). Previous work from our laboratory has confirmed the absence of skMLCK enzyme in this genotype, although no other fiber type differences have been detected (Gittings et al. 2011, 2015). Thus, although we cannot completely eliminate the possibility of unidentified adaptations in the transcriptome of skMLCK^−/−^ muscles, it may be reasonable to assume that the main phenotype difference between wild‐type and skMLCK^−/−^ muscles is the presence and absence, respectively, of RLC phosphorylation. The mechanism for the potentiation displayed by skMLCK^−/−^ muscles may be related to the stimulation‐induced elevations in myoplasmic Ca^2+^ observed in frog skeletal fibers (Cannell 1986; Caputo et al. 1994) and mouse lumbrical muscles (Smith et al. 2013) (c.f. Abbate et al. 2002). Given the possibility that this mechanism may also operate in wild‐type muscles, our data suggest that epinephrine influenced potentiation of mouse EDL muscle via altered Ca^2+^ activation, and not skMLCK catalyzed phosphorylation of the RLC. Unfortunately, our data cannot discern the ability of epinephrine to enhance MLCP phosphatase activity in either genotype, however.

The potentiated twitches of both genotypes displayed enhanced twitch kinetics. The fact that we observed a greater increase in the +dF/dt of wild‐type than skMLCK^−/−^ muscles at virtually every time point in both untreated and treated muscles suggests that RLC phosphorylation was responsible for this outcome. On the other hand, consistent with previous work, the +dF/dt of unpotentiated twitches of both genotypes was increased similarly by epinephrine (Brown et al. 1948; Bowman and Zaimis 1958; Andersson et al. 2012). Interestingly, the epinephrine‐induced increase in +dF/dt tended to rise with time in both genotypes, although it was generally larger in wild‐type than skMLCK^−/−^ muscles (compare Tables 3A and B). Because this effect was similar in both genotypes, it suggests a pathway independent of RLC phosphorylation was responsible, perhaps an effect on Ca^2+^ handling by the sarcoplasmic reticulum (Batts et al. 2007; Liu et al. 1997). Interestingly, the –dF/dt of untreated wild‐type and skMLCK^−/−^ muscles was enhanced for 45–60 sec following stimulation, an effect that was increased by epinephrine. Although the altered kinetics of potentiated isometric twitches has been associated with metabolic accumulation (Smith et al. 2016), it remains unclear how or if this mechanism influences concentric twitches. Finally, the positive influence of epinephrine on twitch kinetics is consistent with an influence on Ca^2+^ handling (e.g., Andersson et al. 2012).

In summary, the present results complement those of Decostre et al. (2000) by showing that the influence of epinephrine on twitch force potentiation of mouse EDL muscle is not mediated via skMLCK. While our experiments did not provide a rigorous test for epinephrine‐induced inhibition of RLC dephosphorylation, they did provide evidence that acute *β*‐adrenergic stimulation enhances potentiation independent of RLC phosphorylation in both genotypes. Thus, although wild‐type muscles display greater potentiation than do skMLCK^−/−^ muscles due to the presence of skMLCK catalyzed RLC phosphorylation, the absence of this signaling pathway does not alter the positive effects of ß‐agonist stimulation on potentiation in either genotype.

## Conflict of Interest

The authors do not have any conflicting interests regarding the findings or interpretations of this manuscript.
